# Microfragmented Adipose Tissue With Adjuvant Platelet-Rich Plasma Combination Therapy for Partial-Thickness Supraspinatus Tear

**DOI:** 10.7759/cureus.15583

**Published:** 2021-06-10

**Authors:** Anuj Marathe, Bo Song, Prathap Jayaram

**Affiliations:** 1 H. Ben Taub Department of Physical Medicine and Rehabilitation, Baylor College of Medicine, Houston, USA

**Keywords:** microfragmented adipose tissue, platelet-rich plasma, supraspinatus, sports medicine, regenerative medicine

## Abstract

A 50-year-old male presented with acute, sharp, right shoulder pain. Ultrasound of the right shoulder revealed a partial thickness tear of the supraspinatus. After conservative management failed to provide any relief, he was treated with microfragmented adipose tissue (MFAT) injection followed by platelet-rich plasma (PRP) at 14 weeks. At the 28-week follow-up, he showed significant improvement in pain and mobility with a resolution of the tear on ultrasound. While PRP has been shown to confer some protection against retears, very few studies have investigated the efficacy of MFAT use in rotator cuff pathology. In this case, we used a combination of MFAT and PRP to successfully treat a partial thickness supraspinatus tear. These agents may function in a synergistic manner, with MFAT providing a cell scaffold and PRP modulating the cellular environment to optimize healing. Further studies are needed to better understand the mechanism of this treatment modality in treating similar conditions.

## Introduction

Rotator cuff tears affect an estimated 25% of individuals in their 60s and 50% of individuals in their 80s [1]. The pathophysiology is multifactorial and includes excess mechanical loads, intrinsic joint anatomy, muscle overuse, and age-related degeneration [2]. Recent studies have shown postsurgical retear rates as high as 13.1% for full-thickness tears, with 40% of asymptomatic partial-thickness tears eventually progressing to full-thickness tears [3,4]. As an alternative to surgical intervention, minimally invasive regenerative strategies such as microfragmented adipose tissue (MFAT) and platelet-rich plasma (PRP) have recently gained popularity [5]. This case highlights the treatment of a partial-thickness supraspinatus tear with combination MFAT and PRP therapy.

## Case presentation

A 50-year-old healthy male with no significant past medical history presented with acute, sharp, right shoulder pain after reaching behind his car seat. On examination, he exhibited significant right shoulder hiking with 8/10 pain on the visual analog scale (VAS) at 70º of abduction and 45º external rotation with limited range of motion (ROM) and weakness when reaching overhead. He also had 4/5 strength during shoulder abduction with positive Neer’s and Hawkin’s tests.

**Figure 1:**
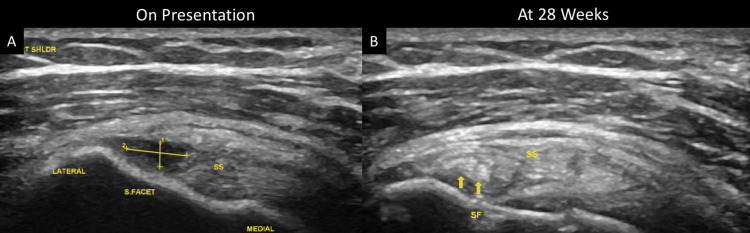
Ultrasound on presentation (A) and at 28 weeks (B). (A) Supraspinatus partial-thickness tear near tendon insertion site measuring 2.5 mm × 6.5 mm. (B) Resolved supraspinatus tear.

Ultrasound of the right shoulder revealed a partial-thickness supraspinatus tear measuring 2.5 mm × 6.5 mm (Figure 1A). After little relief following six weeks of physical therapy focusing on rotator cuff and shoulder strengthening and stretching, he opted for a combination regenerative strategy using MFAT and a subsequent PRP injection. For the MFAT injection, fat was harvested from bilateral gluteal areas and 60 mL of lipoaspirate was collected. The sample was processed through a Lipogems® device (Lipogems International, Milan, Italy), and 3 mL was injected directly into the supraspinatus tendon tear site. He was also restarted on a rotator cuff rehabilitation program one-week post-procedure for 12 weeks. Follow-up at 14 weeks showed moderately improved ROM and pain reduction. At this time, a subsequent PRP injection (5 mL total) was performed into the supraspinatus tear site to further augment the healing process. Follow-up at 28 weeks showed near full recovery of pain (1/10 on VAS) and ROM with complete healing of the supraspinatus tear on ultrasound (Figure 1B).

## Discussion

Mesenchymal stem cells (MSCs) have recently gained traction for their potential applications in regenerative medicine. These cells can be extracted from bone marrow or fat and are capable of multi-lineage differentiation [6]. Traditionally, adipose tissue is enzymatically digested into a stromal vascular fraction (SVF) containing adipose MSCs; however, MFAT uses mild mechanical forces to break up the tissue which preserves the microvascular stroma [7]. Vezzani et al. found that compared to SVF, MFAT has much greater regenerative potential through increased production of cytokines and growth factors responsible for angiogenesis and tissue repair [8]. This is thought to be a result of the intact microvasculature and stromal tissue that allows for better cell scaffolding and long-term MSC survival as well as increased recruitment of CD-163+ wound-healing macrophages compared to SVF [9].

MFAT use has shown promising results in treating knee osteoarthritis (KOA). In fact, it was shown to increase glycosaminoglycan content in existing cartilage even in patients with grade III and IV KOA [10]. Mautner et al. conducted a prospective analysis on patients who received MFAT injections for KOA and found a significant improvement in function and pain [11]. Russo et al. showed similar results in patients with degenerative chondral lesions in the knee who had failed conservative treatment (rehabilitation and other injectables) for at least 12 months [12].

However, there is a severe paucity of clinical data for MFAT use in rotator cuff pathologies. One retrospective animal study examined gait data and tendon ultrasounds of dogs with supraspinatus tendinopathy treated with MFAT plus PRP injection and found significant improvements in gait function and tendon inflammation after 90 days [13]. One human case series by Striano et al. assessed the efficacy of MFAT in rotator cuff tears and shoulder osteoarthritis [14]. Using ultrasound guidance, they directly injected MFAT into tears as well as into the glenohumeral joint space and found statistically significant improvements in pain and function in the short and long term [14]. Additionally, Cherian et al. described one case where MFAT treatment provided complete pain relief for a patient with chronic rotator cuff tear after conservative management had failed [15].

Similar to MFAT, PRP is another biologic agent being studied for its regenerative potential. Platelet alpha-granules contain high levels of growth factors such as platelet-derived growth factor, transforming growth factor-beta, and vascular endothelial growth factor which are believed to help with physiologic wound healing at injected sites [16]. In addition, arthroscopic rotator cuff repairs that used adjuvant PRP had lower retear rates and improved clinical outcomes [17]. Cai et al. also found improved pain and function in patients with small-to-medium partial-thickness rotator cuff tears treated with PRP [18]. Finally, a recent systematic review found significantly lower rotator cuff retear rates in patients treated with PRP [19]. While they also found statistical significance between PRP and placebo in functional scores, they noted that it did not meet the minimum clinical significance criteria [19].

## Conclusions

This case demonstrates the first use of MFAT and PRP combination therapy in a human to treat a partial-thickness supraspinatus tear. Given the mechanisms of both modalities, we suspect a synergistic mechanism. It is likely that the MFAT filled in the torn region, laid a scaffold, and began the repair process, after which PRP further accelerated healing. Surprisingly, the potential synergistic effects of these agents are yet to be studied. Future studies could use standard patient-reported outcome metrics to directly compare efficacy to the current standards of care. While our report is limited to one case, the promising results suggest that utilizing both biologics in combination may lead to better outcomes, especially in rotator cuff tears.
